# CG8005 Mediates Transit-Amplifying Spermatogonial Divisions via Oxidative Stress in *Drosophila* Testes

**DOI:** 10.1155/2020/2846727

**Published:** 2020-10-27

**Authors:** Wanyin Chen, Xiaojin Luan, Yidan Yan, Min Wang, Qianwen Zheng, Xia Chen, Jun Yu, Jie Fang

**Affiliations:** Department of Gynecology, The Affiliated Hospital of Jiangsu University, Jiangsu University, Zhenjiang, Jiangsu 212001, China

## Abstract

The generation of reactive oxygen species (ROS) widely occurs in metabolic reactions and affects stem cell activity by participating in stem cell self-renewal. However, the mechanisms of transit-amplifying (TA) spermatogonial divisions mediated by oxidative stress are not fully understood. Through genetic manipulation of *Drosophila* testes, we demonstrated that CG8005 regulated TA spermatogonial divisions and redox homeostasis. Using in vitro approaches, we showed that the knockdown of CG8005 increased ROS levels in S2 cells; the induced ROS generation was inhibited by NAC and exacerbated by H_2_O_2_ pretreatments. Furthermore, the silencing of CG8005 increased the mRNA expression of oxidation-promoting factors Keap1, GstD1, and Mal-A6 and decreased the mRNA expression of antioxidant factors cnc, Gclm, maf-S, ND-42, and ND-75. We further investigated the functions of the antioxidant factor cnc, a key factor in the Keap1-cnc signaling pathway, and showed that cnc mimicked the phenotype of CG8005 in both *Drosophila* testes and S2 cells. Our results indicated that CG8005, together with cnc, controlled TA spermatogonial divisions by regulating oxidative stress in *Drosophila*.

## 1. Introduction

In adult *Drosophila* testes, two stem cell lineages, germline stem cells (GSCs) and cyst stem cells (CySCs), wrap around hub cells at the apex of the testis to form the germline stem cell niche [1, 2]. Hub cells mainly maintain the self-renewal and differentiation of these two types of stem cells [3, 4]. CySCs differentiate into Cyst cells, which provide an environment for the growth and differentiation of germ cells [2]. GSCs produce two progeny cells: one which stays in the niche to maintain the characteristics of stem cells and the other which departs from the niche, under the relevant signaling, and undergoes transit-amplifying (TA) spermatogonial divisions prior to terminal differentiation [5, 6].

Reactive oxygen species (ROS) are generated as byproducts of various cell metabolism and homeostasis events [7, 8]. An imbalance in ROS generation leads to apoptosis and tissue damage by triggering the caspase cascade [9, 10]. Oxidative stress can affect stem cell behavior by promoting differentiation, proliferation, or apoptosis processes [11]. Evidence shows that mitochondrial dynamics play key roles in regulating early germ cell behavior and are important for regulating the maintenance of early germ cells, in larval testes [12]. By transcriptome analysis of adult testes, 152 genes were found with changes in mRNA expression levels during GSC differentiation induced by ROS accumulation [13]. In the *Drosophila* ovary, a genetic screen for follicle stem cell (FSC) self-renewal is implemented and can identify several genes that are required for FSC maintenance and the regulation of ROS generation. Ectopic expression of catalase or Gpx can eliminate ROS accumulation in mrpL4 and pdsw mutant clones, however, could not rescue defects in FSC maintenance [14].

The Keap1-Nrf2 (Nrf2 also known as cnc in *Drosophila*) signaling transduction pathway has been proven to play highly conserved roles in the regulation of oxidative stress homeostasis [15]. When under a lack of oxidative stress, Nrf2 acts as an antioxidant factor by binding to the cytoplasmic inhibitor Keap1 through Cul3 ligase and preventing its transport into the nucleus. However, when exposed to oxidative stress, Nrf2 relocates from the cytoplasm to the nucleus and combines with Maf proteins to form a heterodimer, where it can recognize and bind with antioxidant response element (ARE) to promote the expression of antioxidant genes [11].

It has been revealed that defects in spermatogonial differentiation caused disorders in spermatogenesis and resulted in the formation of GSC-like tumors in *Drosophila* [16]. Dpp and Gbb proteins, which are recognized as BMP-like molecules, activate the BMP signaling pathway through membrane receptors, thereby inhibiting the expression of the differentiation factor Bam protein [17–19]. In Bam mutant testes, its absence has been shown to cause the accumulation of undifferentiated germ cells and eventually the formation of GSC-like tumors [20, 21]. However, the relationship between TA spermatogonial divisions and oxidative stress remains unknown. Previously, Yu et al. identified CG8005 as a regulator of stem cell niche homeostasis in *Drosophila* testes [22]. In this study, we investigated the actions of CG8005 during TA spermatogonial divisions and explored its potential involvement in the relationship between TA spermatogonial divisions and oxidative stress.

## 2. Materials and Methods

### 2.1. Fly Strains and RNAi Strategies

All flies were raised on standard corn meal food at 25°C and in a relative humidity of 60%. The transgenic RNAi flies used in this study were ordered from the Tsinghua Fly Center (THFC, Beijing, China). Transgenic strains used were as follows: UAS-CG8005 RNAi (THU4150, THFC), UAS-cnc RNAi (1; THU5248, THFC), and UAS-cnc RNAi (2; THU1052, THFC). The Bam-Gal4;*Δ*86/+ line was a gift from DH Chen (Institute of Zoology, Chinese Academy of Sciences, Beijing, China). The W^1118^ line was used as the wild-type (WT) fly.

Male flies of Bam-Gal4 were selected to cross with virgin flies of the UAS-RNAi strains. Selected males with the desired genotypes in their offspring were used for further functional analysis.

### 2.2. Immunofluorescence and Antibodies

Fly testes were dissected in 1x phosphate-buffered saline (PBS) and fixed for 30 min in 4% paraformaldehyde. After washing three times in 1x PBS with 0.1% Triton X-100 (PBST) and blocking for 1 h in 5% bovine serum albumin, the testes were incubated with primary antibodies for 1 h at room temperature. Then, the samples were washed three times for 10 min in 0.1% PBST and incubated for 1 h with secondary antibodies at room temperature followed by the final three washes in 0.1% PBST. Testes were then stained with Hoechst 33342 (1.0 mg/mL; Invitrogen, CA, USA) for 5 min before mounting.

The primary antibodies used in this study were as follows: rabbit anti-Vasa (1 : 200; Santa Cruz Biotechnology, TX, USA), mouse anti-1B1 (1 : 50; Developmental Studies Hybridoma Bank, IA, USA), rat anti-DE-cadherin (1 : 20; DSHB), mouse anti-Eya (1 : 50; DSHB), rat anti-Zfh1 (1 : 1000; a gift from C Tong; Life Sciences Institute, Zhejiang University, Zhejiang, China), rabbit anti-PH3 (1 : 400; Cell Signaling Technology, Leiden, Netherlands), and mouse anti-FasIII (1 : 50; DSHB). Secondary antibodies were conjugated to A488, Cy3, or A647 (Molecular Probes and Jackson Immunological) and used at a dilution of 1 : 1000.

### 2.3. Cell Culture and Transfection

*Drosophila* Schneider 2 (S2) cells were obtained from the *Drosophila* Genomics Resource Center (IN, USA) and cultured in Schneider *Drosophila* Medium (21720024, Gibco, MA, USA) containing 10% heat-inactivated fetal bovine serum (04-001-1ACS; Biological Industries, Israel) at 28°C. The cells were separated with a supplementary medium at a ratio of 1 : 4 every 3–4 days. For the knockdown assay, S2 cells were transfected using Lipofectamine 2000 (Lipo2000; 11668019, Invitrogen). Negative control fragments were transfected and used as controls for S2 cells. siRNAs used in this study were designed and synthesized by GenePharma (Suzhou, China) and are listed in Table S1.

### 2.4. Quantitative Reverse Transcription-Polymerase Chain Reaction (qRT-PCR)

Total RNA was extracted using TRIzol reagent (9108, Takara, Japan), cDNA was synthesized using the PrimeScript RT Reagent Kit (RR037A, Taraka), and qRT-PCR was performed using SYBR Premix Ex Taq (RR420A, Takara), according to the manufacturer's instructions. Glyceraldehyde-3-phosphate dehydrogenase (GAPDH) was used as an internal reference. All primers used for qRT-PCR are listed in Table S2.

### 2.5. ROS Assay

S2 cells were isolated and divided into several groups; NAC or H_2_O_2_ was added to the corresponding group. Samples were incubated at 28°C for 1 h to alter ROS production and then perform negative control and siRNA transfection. Dihydroethidium (DHE; Biyuntian, Shanghai, China) and 2,7-dichlorofluorescein diacetate (DCFH-DA; Biyuntian) were used to detect ROS generation based on fluorescence intensity. DHE fluorescence was used to detect superoxide production, as previously described [23, 24]. Intracellular ROS can oxidize nonfluorescent DCFH to generate fluorescent dichlorofluorescein (DCF) [25]. After transfection for 48 h, cells were incubated with DHE or DCFH-DA probes for 30 min in the dark. After removing DHE or DCFH-DA probes, the cells were washed with PBS. For *Drosophila*, testes were dissected in PBS and incubated with H_2_O_2_ treatment for 1 h at room temperature. After being washed with PBS, the testes were then incubated with DHE working solution in the dark for 5 minutes. After being washed with PBS for three times, the testes were fixed for 30 min in 4% paraformaldehyde and stained with Hoechst 33342 (1.0 mg/mL; Invitrogen, CA, USA) for 5 minutes. Quantification analysis of fluorescence intensity was performed using the ImageJ software.

### 2.6. Statistical Analysis

Each experiment was repeated at least three times, and the data are presented as the mean ± standard error of the mean (SEM). Comparisons among groups were determined by one-way analysis of variance (ANOVA) and Student' s *t*-test using the GraphPad Software (https://www.graphpad.com/).

## 3. Results

### 3.1. CG8005 Is Required for TA Spermatogonial Divisions

**Figure 1:**
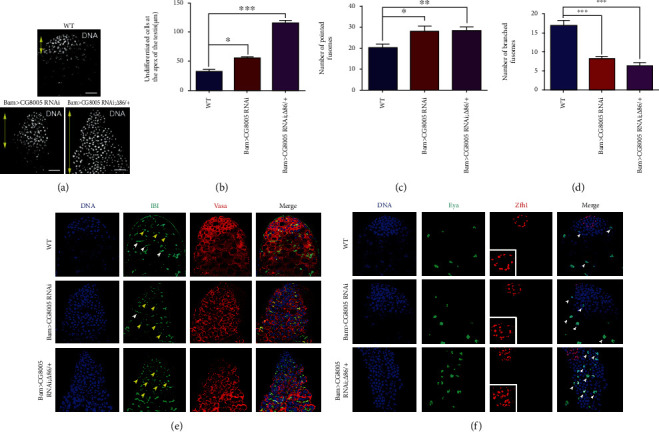
Knockdown of CG8005 in spermatogonia resulted in differentiation defects. (a) DNA staining in WT, Bam>CG8005 RNAi, and Bam>CG8005 RNAi;*Δ*86/+ testes. Yellow double arrows label undifferentiated cells at the apex of the testis. (b) The distance of undifferentiated cells at the apex of the testis. (c) The number of pointed fusomes. (d) The number of branched fusomes. (e, f) Immunostaining of WT, Bam>CG8005 RNAi, and Bam>CG8005 RNAi;*Δ*86/+ testes. Representative pointed fusomes are indicated with yellow arrowheads, and branched fusomes are indicated with white arrowheads. ^∗^*P* < 0.05, ^∗∗^*P* < 0.01, and ^∗∗∗^*P* < 0.001. Scale bar: 20 *μ*m.

To examine the physiological function of CG8005 during TA spermatogonial division, we performed a CG8005 RNAi assay in spermatogonia derived from Bam-Gal4 flies. It has been previously reported that the deletion of Bam, a key differentiation factor, could lead to defects in germ cell differentiation [26]. At the apex of the testis, the early stage of germ cells and cyst cells can be stained by DNA dye. Compared to WT flies, undifferentiated cells accumulated in Bam>CG8005 RNAi testes, and the phenotype was enhanced by heterozygous mutation of Bam in Bam>CG8005 RNAi testes (Figures 1(a) and 1(b)). In WT testes, fusomes, which were labeled by 1B1, exhibited dynamic changes with punctate distribution in the early stage of germ cells, followed by development and transition into branched shapes in differentiated germ cells [27]. Then, we used immunofluorescence staining to analyze the apex of testes of both WT and CG8005 RNAi flies. In Bam>CG8005 RNAi testes, the amount of accumulated undifferentiated cells, labeled by Vasa, and pointed fusomes dramatically increased, while the number of branched fusomes significantly decreased (Figures 1(c), 1(d), and 1(e)). Moreover, the differentiation defects of early stage germ cells were clearly exacerbated in Bam>CG8005 RNAi;*Δ*86/+ testes (Figures 1(c), 1(d), and 1(e)). Phosphorylated histone H3 (phospho-histone H3, PH3) was used as a marker for proliferating cells. We further used immunofluorescence technology to detect whether these accumulated undifferentiated cells obtained proliferation ability. FasIII was used to recognize the hub cells at the apex of the testis. In this study, we found that undifferentiated germ cells obtained their self-renewal ability without normal niche signals (Figure S1). We also stained testes with eyes absent (Eya) and zn finger homeodomain 1 (Zfh1) to distinguish mature cyst cells and early stage cyst cells, respectively. Interestingly, mature cyst cells and early stage cyst cells were present in both Bam>CG8005 RNAi and Bam>CG8005 RNAi;*Δ*86/+ testes (Figure 1(f)). Taken together, our results indicated that CG8005 mediated germ cell differentiation in spermatogonia.

### 3.2. Inhibition of CG8005 Increased ROS Levels in Drosophila Testes

**Figure 2:**
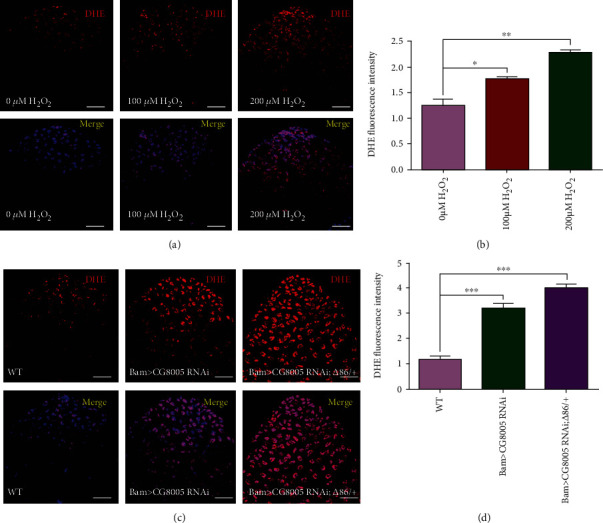
ROS generation in CG8005 RNAi testes. (a) DHE staining in testes with H_2_O_2_ treatment. (b) DHE fluorescence intensity of testes with H_2_O_2_ treatment. (c) DHE staining in WT, Bam>CG8005 RNAi, and Bam>CG8005 RNAi;*Δ*86/+ testes. (d) DHE fluorescence intensity of WT, Bam>CG8005 RNAi, and Bam>CG8005 RNAi;*Δ*86/+ testes. ^∗^*P* < 0.05, ^∗∗^*P* < 0.01, and ^∗∗∗^*P* < 0.001. Scale bar: 20 *μ*m.

ROS signals are considered important stem cell regulators and affect stem cell homeostasis by facilitating the differentiation and self-renewal of multiple stem cell populations [28]. To further investigate whether dysfunctional CG8005-mediated spermatogonial differentiation defects were involved in oxidative stress, we examined the effect of the redox state on both WT and CG8005 RNAi testes. Hydrogen peroxide (H_2_O_2_) is a recognized oxidant, and we determined ROS levels by DHE intensity in H_2_O_2_-treated testes. We found that ROS production increased with H_2_O_2_ dosage-dependent effects on *Drosophila* testes (Figures 2(a) and 2(b)). Importantly, we also found that ROS production dramatically increased in both Bam>CG8005 RNAi and Bam>CG8005 RNAi;*Δ*86/+ testes compared with WT testes (Figures 2(c) and 2(d)), indicating that CG8005 is required for redox homeostasis in *Drosophila* testes.

### 3.3. CG8005 Inhibited ROS Levels in S2 Cells

**Figure 3:**
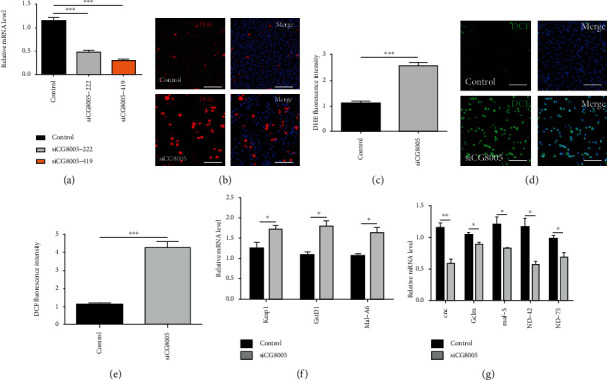
Knockdown of CG8005 induced ROS accumulation in S2 cells. (a) RNAi efficiency verification of CG8005 in control and siCG8005 (siCG8005-222 and siCG8005-419) S2 cells. (b) DHE staining in control and siCG8005 S2 cells. (c) DHE fluorescence intensity in control and siCG8005 S2 cells. (d) DCF staining in control and siCG8005 S2 cells. (e) DCF fluorescence intensity in control and siCG8005 S2 cells. (f) Relative mRNA levels of oxidation-promoting factors (Keap1, GstD1, and Mal-A6) in control and siCG8005 S2 cells. (g) Relative mRNA levels of antioxidant factors (cnc, Gclm, maf-S, ND-42, and ND-75) in control and siCG8005 S2 cells. ^∗^*P* < 0.05, ^∗∗^*P* < 0.01, and ^∗∗∗^*P* < 0.001. Scale bar: 30 *μ*m.

To further analyze the function of CG8005 by an in vitro approach, we used two small interfering RNAs (siCG8005-222 and siCG8005-419) to silence the CG8005 gene. The relative mRNA expression level of CG8005 was examined in S2 cells, and the results showed that the knockdown efficiency of siCG8005-419 was the highest and most stable at a concentration of 150 nM (Figure 3(a)). Since ROS levels were upregulated in CG8005 RNAi testes, we further tested ROS production by using both DHE and DCF probes, and the results demonstrated that both DHE and DCF fluorescence intensities were dramatically increased in siCG8005-treated S2 cells (Figures 3(b)–3(e)). Furthermore, we detected the relative mRNA levels of oxidative stress-related factors in S2 cells and found a significant increase in the mRNA expression of oxidation-promoting factors Keap1, GstD1, and Mal-A6 and a significant decrease in the mRNA expression of antioxidant factors cnc, Gclm, maf-S, ND-42, and ND-75, as validated by qRT-PCR in control and siCG8005-treated S2 cells (Figures 3(f) and 3(g)). Together, these results supported the notion that CG8005 mediated ROS levels via oxidative stress signals.

### 3.4. Antioxidant Treatments with NAC Inhibited siCG8005-Mediated ROS Accumulation in S2 Cells

**Figure 4:**
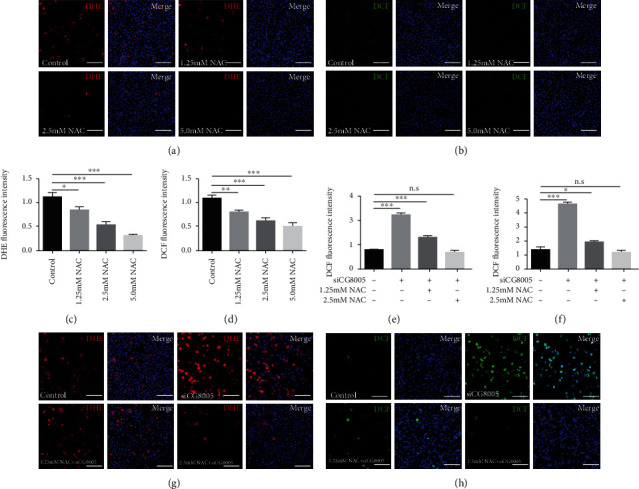
Pretreatment with NAC in siCG8005-mediated S2 cells. (a, b) DHE (a) and DCF (b) staining in control, 1.25 mM NAC, 2.5 mM NAC, and 5.0 mM NAC-treated S2 cells. (c, d) DHE (c) and DCF (d) fluorescence intensities in control, 1.25 mM NAC-, 2.5 mM NAC-, and 5.0 mM NAC-treated S2 cells. (e, f) DHE (e) and DCF (f) fluorescence intensities of pretreatment with NAC in siCG8005-treated S2 cells. (g, h) DHE (g) and DCF (h) staining of pretreatment with NAC in siCG8005-treated S2 cells. ^∗^*P* < 0.05, ^∗∗^*P* < 0.01, and ^∗∗∗^*P* < 0.001. Scale bar: 30 *μ*m.

NAC is an antioxidant commonly used to inhibit intracellular ROS production. Under the antioxidant treatments with NAC, ROS production, as detected by DHE and DCF probes, was greatly decreased with dosage-dependent effects in S2 cells (Figures 4(a)–4(d)). Next, we investigated the effects of NAC on siCG8005-treated S2 cells. Pretreatment with dose-dependent NAC eliminated the accumulation of ROS induced by the siCG8005-mediated oxidative stress imbalance, and antioxidant treatment with NAC at a final concentration of 2.5 mM was sufficient to recover the ROS levels in siCG8005-treated S2 cells (Figures 4(e)–4(h)). These results indicated that NAC prevented the imbalance of intracellular ROS attributed to CG8005 knockdown in S2 cells.

### 3.5. Pretreatment with H_2_O_2_ Aggravated siCG8005-Induced ROS Production in S2 Cells

**Figure 5:**
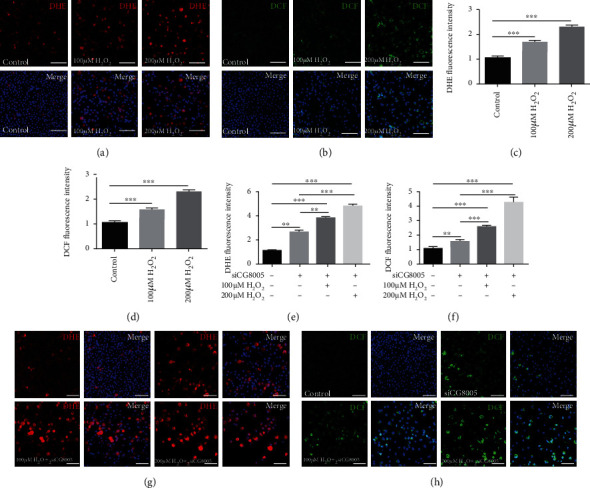
Pretreatment with H_2_O_2_ in siCG8005-mediated S2 cells. (a, b) DHE (a) and DCF (b) staining in control, 100 *μ*M H_2_O_2_-treated, and 200 *μ*M H_2_O_2_-treated S2 cells. (c, d) DHE (c) and DCF (d) fluorescence intensities in control, 100 *μ*M H_2_O_2_-treated, and 200 *μ*M H_2_O_2_-treated S2 cells. (e, f) DHE (e) and DCF (f) fluorescence intensities of pretreatment with H_2_O_2_ in siCG8005-treated S2 cells. (g, h) DHE (g) and DCF (h) staining of pretreatment with H_2_O_2_ in siCG8005-treated S2 cells. ^∗∗^*P* < 0.01 and ^∗∗∗^*P* < 0.001. Scale bar: 30 *μ*m.

We investigated the effects of pretreatment with H_2_O_2_ using DHE and DCF probes in siCG8005-treated S2 cells. First, pretreatment with H_2_O_2_ (at 100 *μ*M and 200 *μ*M) elevated DHE and DCF fluorescence intensities, reflecting ROS dosage-dependent effects in S2 cells (Figures 5(a)–5(d)). Moreover, pretreatment with dose-dependent H_2_O_2_ increased the accumulation of ROS and significantly elevated the fluorescence intensity of DHE and DCF in siCG8005-treated S2 cells (Figures 5(e)–5(h)). Together, these data suggested that pretreatment of H_2_O_2_ in S2 cells could not reduce and instead further aggravated oxidative stress induced by the silencing of CG8005.

### 3.6. Antioxidant Factor cnc Is Required for TA Spermatogonial Divisions and ROS Production in Drosophila Testes

**Figure 6:**
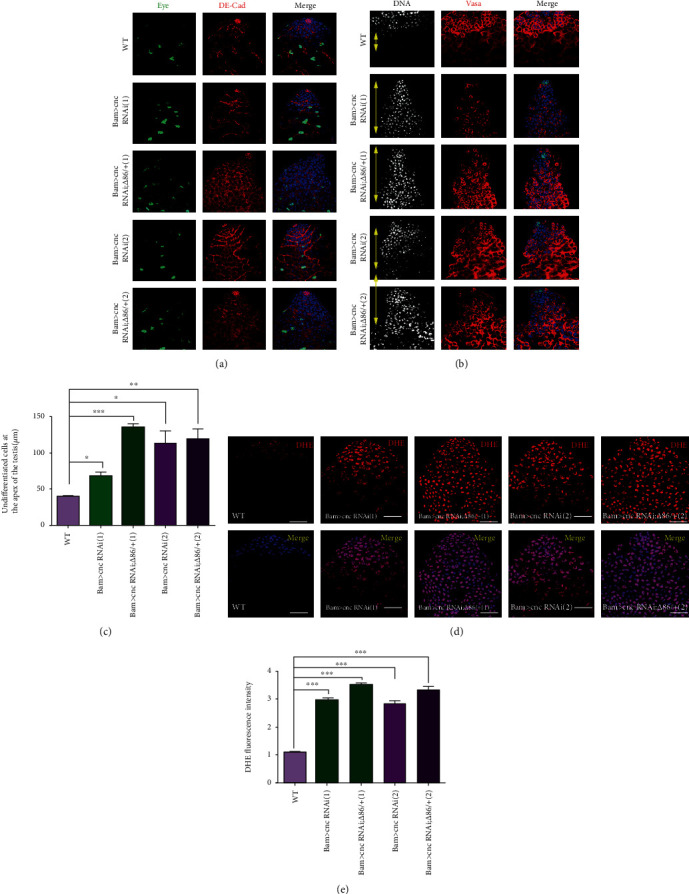
Knockdown of cnc in spermatogonia led to differentiation defects and ROS accumulation in *Drosophila* testes. (a, b) Immunostaining of WT, Bam>cnc RNAi, and Bam>cnc RNAi;*Δ*86/+ testes. Two independent UAS-cnc RNAi lines were used for the functional analysis of cnc. Yellow double arrows label undifferentiated cells at the apex of the testis. (c) The distance of undifferentiated cells at the apex of the testis. (d) DHE staining in WT, Bam>cnc RNAi, and Bam>cnc RNAi;*Δ*86/+ testes. (e) DHE fluorescence intensity of WT, Bam>cnc RNAi, and Bam>cnc RNAi;*Δ*86/+ testes. ^∗^*P* < 0.05, ^∗∗^*P* < 0.01, and ^∗∗∗^*P* < 0.001. Scale bar: 20 *μ*m.

The Keap1-cnc signaling pathway is highly conserved in a variety of cells and tissues as an antioxidant defense. Previous studies have indicated that cnc is an antioxidant factor that interacts with the product of Keap1 to regulate oxidative stress. To determine the role of cnc, we used two independent cnc RNAi, UAS-cnc RNAi (1) and UAS-cnc RNAi (2), driven by Bam-Gal4 to knock down cnc in *Drosophila* testes. As predicted, the knockdown of cnc in spermatogonia did not affect the survival of cyst cells (Figure 6(a)). To explore the effects of cnc on spermatogonial differentiation, we stained testes with DNA and Vasa and found that undifferentiated germ cells accumulated in both Bam>cnc RNAi and Bam>cnc RNAi;*Δ*86/+ testes (Figures 6(b) and 6(c)). Moreover, the degree of differentiation defects was strengthened by heterozygous mutation of Bam (*Δ*86/+) in Bam>cnc RNAi testes (Figures 6(b) and 6(c)). To further investigate the functions of CG8005 on redox balance, we assessed ROS production using a DHE probe. Interestingly, we found that ROS levels were dramatically upregulated in both Bam>cnc RNAi and Bam>cnc RNAi;*Δ*86/+ testes compared with the WT testes (Figures 6(d) and 6(e)), which mimicked the phenotype of CG8005 in *Drosophila* testes. Taken together, these data indicated that cnc contributed to controlling spermatogonial differentiation via ROS in *Drosophila* testes.

### 3.7. Antioxidant Factor cnc Regulated Oxidative Stress in S2 Cells

**Figure 7:**
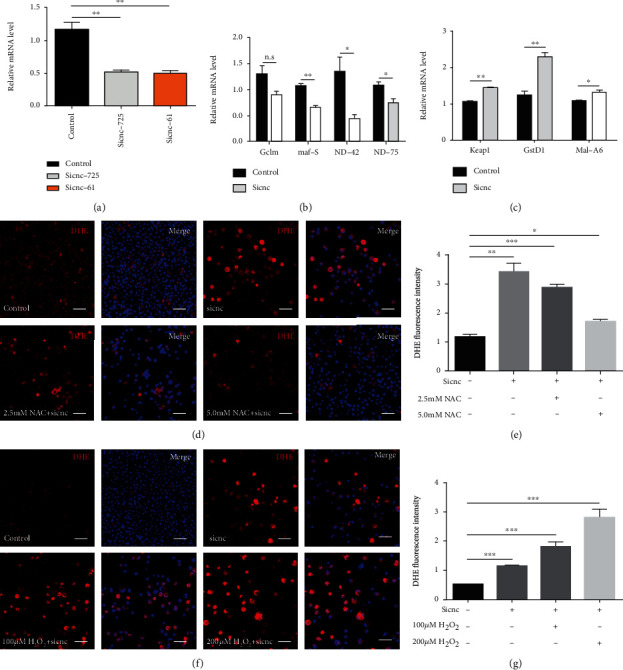
Effects of ROS accumulation in sicnc-treated S2 cells. (a) RNAi efficiency verification of cnc in control and cnc-siRNA (sicnc-725 and sicnc-61) S2 cells. (b) Relative mRNA levels of antioxidant factors (Gclm, maf-S, ND-42, and ND-75) in control and sicnc S2 cells. (c) Relative mRNA levels of oxidation-promoting factors (Keap1, GstD1, and Mal-A6). (d, e) DHE staining (d) and DHE fluorescence intensity (e) of pretreatment with NAC in sicnc-treated S2 cells. (f, g) DHE staining (f) and DHE fluorescence intensity (g) of pretreatment with H_2_O_2_ in sicnc-treated S2 cells. ^∗^*P* < 0.05, ^∗∗^*P* < 0.01, and ^∗∗∗^*P* < 0.001. Scale bar: 30 *μ*m.

To further analyze the roles of cnc in S2 cells, we silenced the cnc gene using two siRNAs (sicnc-725 and sicnc-61) and qRT-PCR was used to verify the interference efficiency (Figure 7(a)). Additionally, the mRNA expression of antioxidant factors maf-S, ND-42, and ND-75 was significantly decreased, and the mRNA expression of oxidation-promoting factors Keap1, GstD1, and Mal-A6 was dramatically increased in sicnc-treated S2 cells (Figures 7(b) and 7(c)).

We also used DHE and DCF probes to detect the state of oxidative stress and found that ROS production was increased in sicnc-treated S2 cells (Figures 7(d)–7(g) and Figure S2). Since cnc is essential for spermatogonial differentiation via ROS generation, we evaluated antioxidant and oxidant treatment in sicnc-treated S2 cells. Pretreatment with dose-dependent NAC partially reduced the accumulation of ROS in sicnc-treated S2 cells (Figures 7(d) and 7(e) and Figures S2(a) and S2(b)). Moreover, pretreatment with dose-dependent H_2_O_2_ increased ROS levels and significantly elevated the fluorescence intensity of DHE and DCF in sicnc-treated S2 cells (Figures 7(f) and 7(g) and Figures S2(c) and S2(d)). Taken together, these results demonstrated that the antioxidant factor cnc was essential for redox homeostasis.

## 4. Discussion

In *Drosophila* testes, hub cells maintain the normal behavior of GSCs and CySCs in the niche environment, and GSCs possess strong self-renewal and differentiation abilities [29]. To produce sufficient differentiated progeny for germline homeostasis, the gonialblast undergoes multiple rounds of TA divisions prior to terminal differentiation [4, 30]. TA spermatogonial divisions are particularly important for normal spermatogenesis, and failure to exit the TA divisions may cause overgrowth during spermatogonial differentiation [6]. A previous report has shown that the mutated CG32364 gene (designated as tut), which encodes a putative RNA-binding protein, acts alongside Bam and Bgcn to form the Tut-Bam-Bgcn complex and represses the translation of mei-P26 mRNA to effectively regulate the balance of proliferation and differentiation in TA cells [31]. CG8005 is identified as a regulator of GSCs in *Drosophila* testes, although its mechanisms have yet to be revealed. In this study, we provided evidence for the significant roles and mechanisms of CG8005 and cnc during TA spermatogonial divisions. Our data suggested that loss of CG8005 and cnc in spermatogonia resulted in the accumulation of undifferentiated germ cells. These differentiation defects were further enhanced by heterozygous mutation of the differentiation factor Bam, indicating that both CG8005 and cnc were involved in regulating spermatogonial TA divisions.

Oxidative stress is involved in various human diseases such as neurodegenerative diseases, cardiovascular diseases, type II diabetes, and cancer [32–34]. The proliferation, differentiation, and division of various stem cell populations are interfered with by the disruption of redox homeostasis [13, 35]. High levels of ROS have been found in a variety of tissue damage, and oxidative stress has been reported to be necessary for both stem cell maintenance and proliferation [36]. CG8005, a potential deoxythreonate synthase, catalyzed the NAD-dependent oxidative cleavage of spermidine and interacted with eEF5 protein, which is involved in cell cycle progression, mRNA decay, the stress response, and maintenance of the cell wall integrity (http://flybase.org). The potential role of many antioxidants, such as NAC, to improve ROS-related stem cell self-renewal and differentiation disorders has been studied [37, 38]. H_2_O_2_ can exhibit its effect on cells through oxidative stress, which causes cells to gradually lose their ability to proliferate and eventually leads to cell death [39, 40]. In this study, knockdown of CG8005 and cnc induced high levels of ROS generation, which were eliminated by pretreatment with NAC or enhanced by pretreatment with H_2_O_2_. We also found that the antioxidants CG8005 and cnc could regulate the expression of oxidative and antioxidative factors.

## 5. Conclusion

In summary, the present study showed that CG8005 participated in the switch from self-renewal to differentiation during TA spermatogonial divisions and regulated oxidative stress via the Keap1-cnc signaling pathway. Pretreatment with NAC decreased ROS accumulation in CG8005- and cnc-siRNA-mediated S2 cells, while the opposite effect occurred in H_2_O_2_-treated S2 cells. The findings of this study provide new insights for understanding the relationships between TA spermatogonial divisions and oxidative stress.

## Data Availability

The data used to support the findings of this study are available from the corresponding author upon request.

## Supplementary Materials

Supplementary Materials Figure S1: proliferation analysis in CG8005 RNAi testes. Figure S2: effects of pretreatment with NAC and H_2_O_2_ in cnc-siRNA-mediated S2 cells. Table S1: siRNA sequences used in this study. Table S2: primer sequences used in this study.
