# Supplementary material for: CG8005 Mediates Transit-Amplifying Spermatogonial Divisions via Oxidative Stress in Drosophila Testes
Source: Oxid Med Cell Longev. 2020 Oct 27;2020:2846727. doi: 10.1155/2020/2846727 (PMC7641671; doi:10.1155/2020/2846727)
Supplement: Supplementary Materials — Figure S1: proliferation analysis in CG8005 RNAi testes. Figure S2: effects of pretreatment with NAC and H2O2 in cnc-siRNA-mediated S2 cells. Table S1: siRNA sequences used in this study. Table S2: primer sequences used in this study. [file 2846727.f1.docx]

**Supplementary Materials**


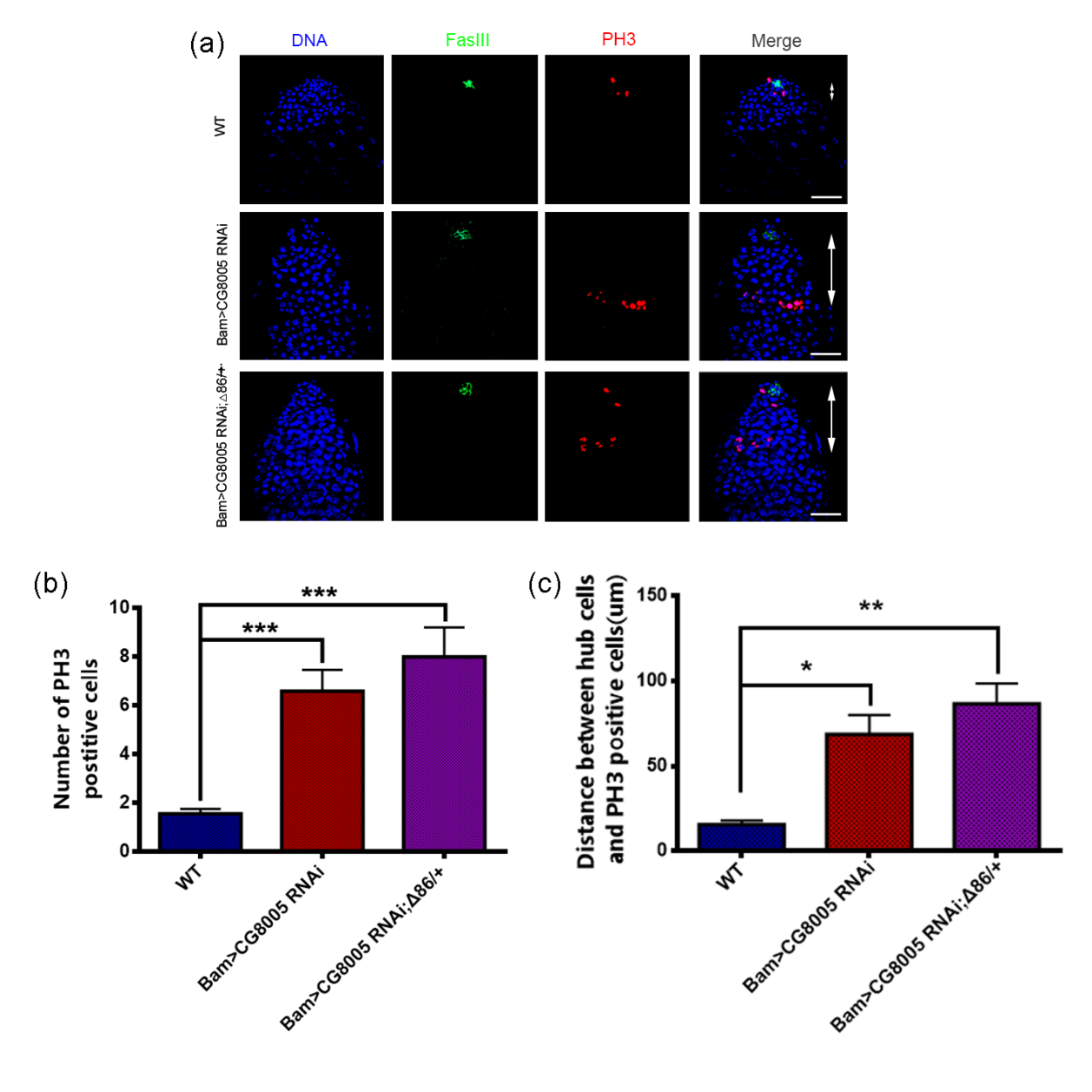


Figure S1: Proliferation analysis in CG8005 RNAi testes. (a) Immunostaining of PH3 (red) and FasIII (green) in WT, Bam>CG8005 RNAi, and Bam>CG8005 RNAi; Δ86/+ testes. White double arrows indicate distance between hub cells and PH3 positive cells. (b) Number of PH3 positive cells in WT, Bam>CG8005 RNAi, and Bam>CG8005 RNAi; Δ86/+ testes. (c) Distance between hub cells and PH3 positive cells in WT, Bam>CG8005 RNAi, and Bam>CG8005 RNAi; Δ86/+ testes. * P < 0.05, **P < 0.01, and ***P < 0.001. Scale bar: 20 µM.


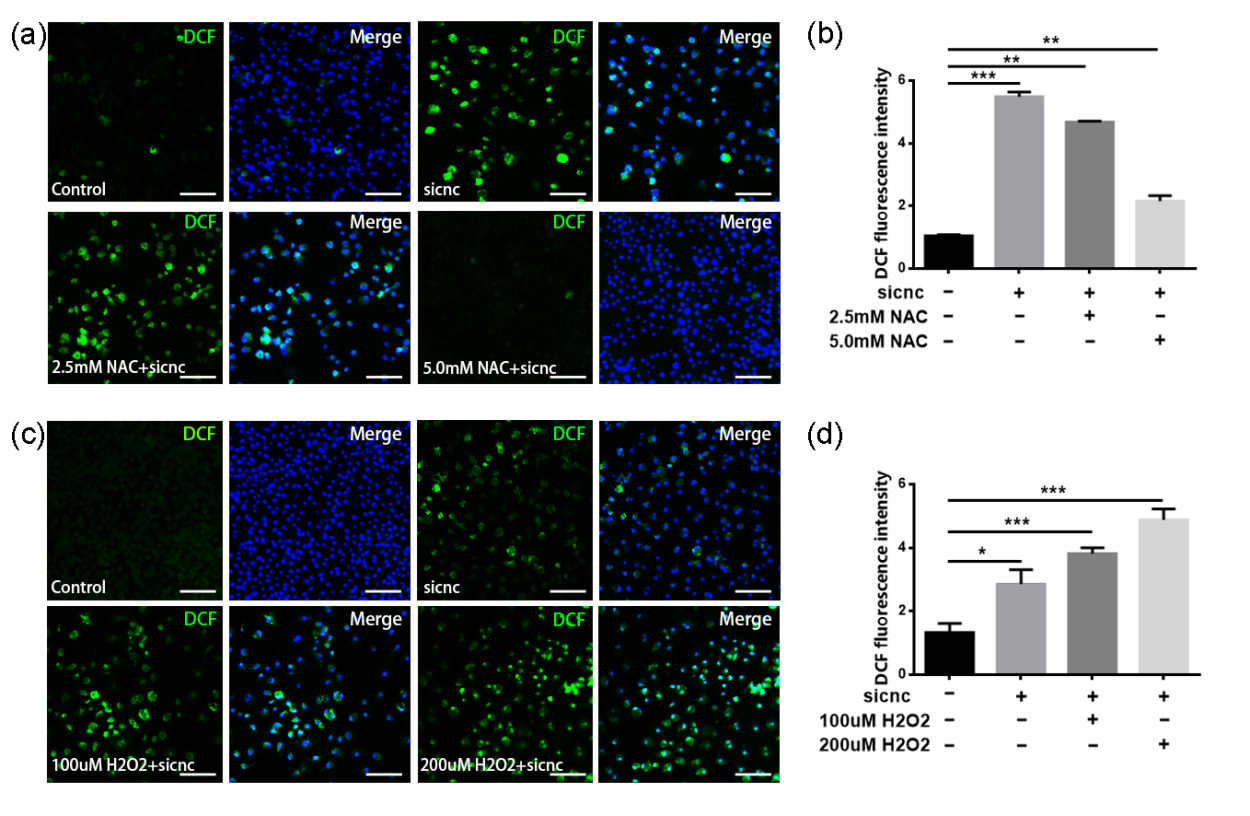


Figure S2: Effects of pretreatment with NAC and H_2_O_2_ in sicnc-treated S2 cells. (a–b) DCF staining (a) and DCF fluorescence intensity (b) of pretreatment with NAC in sicnc-treated S2 cells. (c–d) DCF staining (c) and DCF fluorescence intensity (d) of pretreatment with H_2_O_2_ in sicnc-treated S2 cells. *P < 0.05, **P < 0.01, and ***P < 0.001. Scale bar: 30 µm.

TABLE S1: siRNA sequences used in this study.

| Gene | Forward primer (5'–3') | Reverse primer (5'–3') | |
| --- | --- | --- | --- |
| Control | UUCUCCG AACGUGUCACGUTT | | ACGUGACACGUUCGGAGAATT |
| siCG8005-419 | CCACGUUCAUGGGUUCAUUTT | | AAUGAACCCAUGAACGUGGTT |
| siCG8005-222 | GGACCAAAUAGACAGCCAUTT | | AUGGCUGUCUAUUUGGUCCTT |
| sicnc-61 | GCAGUGGACAUGUUAGCAATT | | UUGCUAACAUGUCCACUGCTT |
| sicnc-725 | GCCAAGGACUUCGCAGGAUTT | | AUCCUGCGAAGUCCUUGGCTT |

TABLE S2: Primer sequences used in this study.

| Gene | Forward primer(5'–3') | Reverse primer(5'–3') |
| --- | --- | --- |
| Keap1 | GCACAAGTGTCCAGGAGTCAAGG | GCCAGTGTCGTCCACGTCATG |
| Gclm | CACCGCTCACGACATCCAACTG | AACGCAGTGTCCAGTCGATTGTG |
| maf-S | TGCCGCATCAAGAGAATCGAACAG | GCTCGTTGTCCTCGTGCATCTG |
| ND-42 | GAAGCAGATCAAGGCACGCAATG | TCCTCCACCACAACCTCAGTCTC |
| ND-75 | TCTGTCCTACGACCACGAGAACC | CACCATCGGCACGCTCCAAC |
| GstD1 | TGGCAACCGTGTCCACATTCG | CACCTTCTTGGCGTTCTCGTACC |
| Mal-A6 | TCCTCGCTCAAGAGTCACAGTCG | ACGCACGCTCGCTTATGGTTG |
| CG8005 | CTGGACTCGTGGTGGACATTCTG | ACACTGAGTAATCCGCTCCATTGC |
| cnc | GGAGATGACGAGGAGGAGAGTGAG | CCGCTGGCATAGGAGGCATTG |
| GAPDH | GTGGTGAACGGCCAGAAGAT | GCCTTGTCAATGGTGGTGAA |
